# The effect of mind–body exercise on cognitive function and neuroplasticity in elderly people with mild cognitive impairment: a systematic review and meta-analysis

**DOI:** 10.3389/fnagi.2025.1683808

**Published:** 2025-12-05

**Authors:** Huifang Tian, Xi Yang, Jiahuan Li, Yuqi Cheng, Shui Tian, Fanfan Meng, Qinqin Zhu, Ying Shen, Tong Wang, Chuan Guo, Yi Zhu

**Affiliations:** 1 Department of Rehabilitation Medicine, The First Affiliated Hospital of Nanjing Medical University, Nanjing, China; 2 The Fourth Affiliated Hospital of Soochow University (Suzhou Dushu Lake Hospital), Suzhou, China; 3 Department of Radiology, The First Affiliated Hospital of Nanjing Medical University, Nanjing, China

**Keywords:** mind–body exercise, mild cognitive impairment, gray matter volume, resting state magnetic resonance, event-related potential

## Abstract

**Objective:**

This systematic review and meta-analysis aims to comprehensively analyze the effects of mind–body exercise on cognitive function, brain structure, and brain function in individuals with mild cognitive impairment (MCI) by assessing randomized controlled trials.

**Methods:**

A systematic search was conducted using four databases: Cochrane Library, EMBASE, PubMed, and Web of Science, from inception until December 2023. The study quality was assessed using the Cochrane risk-of-bias tool. Systematic review and meta-analyses were performed for outcome measures such as the Montreal Cognitive Assessment (MoCA), gray matter volume (GMV), functional connectivity at rest (rsFC), amplitude of low-frequency fluctuation (ALFF) and event-related potential (ERP) P300 latency. Three-dimensional coordinates of brain regions with notable variances were extracted from imaging and delineated in the brain map.

**Results:**

After screening 433 studies, nine met the eligibility inclusion criteria. In 4 studies using the MoCA scale, meta-analysis showed a significant effect of aerobic exercise intervention on global cognitive function improvement (MD = 1.6; 95% CI: 0.70 to 2.50; *p* = 0.0005). Most of the included studies reported that mind–body exercise improved gray matter volume in the hippocampus, bilateral anterior cingulate gyrus, frontotemporo-occipital regions, altered functional connectivity of the default mode network (DMN) and dorsal attentional network (DAN), neural activity in key brain regions in older adults with MCI.

**Conclusion:**

This systematic review demonstrates that mind–body exercise is associated with improved cognitive function and neuroplastic changes in older adults with mild cognitive impairment, with changes particularly evident in regions vulnerable to neurodegeneration such as the hippocampus and anterior cingulate cortex.

**Systematic review registration:**

CRD42022251115; https://www.crd.york.ac.uk/PROSPERO/view/CRD42022251115.

## 1 Introduction

Mild cognitive impairment (MCI) is an intermediate stage between regular aging and dementia and serves as a crucial risk factor for the progression to Alzheimer’s disease (AD) (Xue et al., 2018; Jia et al., 2021). The prevalence of MCI increases with age, with estimates ranging from 6.7 to 25.2% among older adults (Petersen et al., 2018). Individuals with MCI experienced conversion rates of 28% to AD (Hu et al., 2017). Over 50 million people worldwide suffer from dementia, and this number will reach 152 million by 2050 (Jia et al., 2021; Porsteinsson et al., 2021). However, existing AD therapies have limited effectiveness. Initiating effective therapies at the MCI stage is therefore vital to delay the progression of the disease and alleviate the associated economic and caregiving burdens (Zhang et al., 2021).

Until now, there has been a lack of drug treatments shown to effectively delay the progress from MCI to dementia (Anderson, 2019). The recent approval of disease-modifying therapies, particularly anti-amyloid-β monoclonal antibodies, represents a pivotal advance in Alzheimer’s disease therapeutics. Large-scale clinical trials have demonstrated their capacity to reduce cerebral amyloid burden and slow cognitive and functional decline in patients with early-stage Alzheimer’s disease (Qiao et al., 2024; Tonegawa-Kuji et al., 2025). However, substantial limitations remain, including high treatment costs, the risk of amyloid-related imaging abnormalities, and unconfirmed efficacy in individuals with MCI of heterogeneous or non-AD etiologies (Wu et al., 2023; Qiao et al., 2024). These constraints underscore the importance of developing complementary intervention strategies.

Recent studies suggest that certain lifestyle changes and non-pharmacological interventions, such as adopting a Mediterranean diet, engaging in regular exercise, and participating in cognitive training, could be effective (Petersen et al., 2014, 2018). Research indicates that physical activity may modulate brain-derived neurotrophic factor (BDNF), boost neural plasticity, and mitigate age-related brain atrophy, particularly in brain areas linked to dementia such as the hippocampus and frontal temporal lobe (de Sousa Fernandes et al., 2020; Domingos et al., 2021). Multi-component exercise is frequently the most effective in preserving global cognition (Huang et al., 2022a).

Mind–body exercises are classified as multi-component exercises that highlight the harmony of physical movement, mindfulness, and controlled breathing. Numerous studies have demonstrated the beneficial effects of mind–body exercises, including Tai Chi, Baduanjin, yoga, and dance, on overall cognitive function, visuospatial memory, and executive function (Wang et al., 2021; Yuan et al., 2022; Yao et al., 2023; Chen et al., 2024; Giridharan et al., 2024). The human brain is susceptible to changes from external and internal factors, including physical activity, this plasticity persists into adulthood (Colcombe et al., 2006). Harnessing this trait of the brain, a growing number of researchers promote neuroplasticity through mind–body exercise to improve cognitive abilities (Colcombe et al., 2006; Chen et al., 2020; Domingos et al., 2021). Coordination, another hallmark of mind–body exercise, also promotes neuroplasticity, and high coordination, complexity and novelty may be key to cognitive benefits (Kosmat and Vranic, 2017). It has the potential to stimulate various cognitive domains simultaneously (Chan et al., 2020), and increases the volume of brain regions such as the hippocampus, cingulate cortex and insula (Rehfeld et al., 2018; Gothe et al., 2019; Teixeira-Machado et al., 2019; Krause-Sorio et al., 2022). Additionally, mind–body exercise plays a crucial role in fostering interhemispheric connectivity in the brain, bolstering neural activation in motor, sensory, and cognitive regions (Rehfeld et al., 2018).

The potential of mind–body exercises has been gradually uncovered; however, most current systematic reviews concentrate on individual interventions, such as Baduanjin, yoga, or dance, lacking thorough analyses of various forms of mind–body exercises. These diverse mind–body exercises share a common theoretical foundation in mind–body integration. Their practice requires the coordinated engagement of a complex set of components, such as movement, balance, breath control, and sustained attention. This process fosters neuroplasticity through the dynamic interaction of sensory perception, motor execution, and cognitive processing. Moreover, the majority of studies have primarily examined subjective outcome indicators without adequately incorporating evidence on neuroplasticity mechanisms. Therefore, this systematic review and meta-analysis employs a comprehensive approach to evaluate the effects of various mind–body exercises on cognitive function and neuroplasticity in individuals with MCI. By identifying the consistent patterns of brain changes induced by these interventions, this work aims to highlight promising targets for future mechanistic studies and inform the development of evidence-based interventions.

## 2 Methods

This study was constructed according to the guidelines for Preferred Reporting Items for Systematic Review and Meta-Analysis (PRISMA) (Moher et al., 2009). The review programme has been registered with the International System Review Prospective Registry (CRD42022251115).

### 2.1 Literature retrieval

A systematic literature search was performed independently by two investigators across four electronic databases: PubMed, EMBASE, the Cochrane Library, and Web of Science. The search was conducted from database inception until December 2023. A secondary search of the included references was conducted to find the studies that met the inclusion criteria as comprehensively as possible. The search strategy incorporated a combination of keywords related to three key domains: (1) Disease type search terms: mild cognitive impairment, MCI. (2) Intervention methods search terms: dancing, dance, social dance, ballroom dance, classical dance, aerobic dance, disco, salsa waltz, latin and tai chi. (3) Outcome measures search terms: brain structure, brain function, brain plasticity, MRI, magnetic resonance imaging, functional magnetic resonance imaging, fMRI, grey matter, white matter, Diffusion Tensor Imaging (DTI), functional connectivity.

### 2.2 Inclusion and exclusion criteria

The study followed the PICOS principles: (1) Population: Participants needed to be aged 50 years or older with a formal diagnosis of Mild Cognitive Impairment (MCI). (2) Interventions: The interventions comprised structured mind–body exercise programs, including diverse dance forms (like ballroom dance, waltz, Latin dance, classical dance, aerobic dance) and traditional practices such as Tai Chi and Baduanjin. (3) Comparator: Control groups received either health education sessions, engaging in repetitive physical exercises (e.g., brisk walking), or a combination of both, but did not participate in any mind–body exercise training. (4) Outcomes: Primary outcome measures included the Montreal Cognitive Assessment (MoCA) for global cognitive function, gray matter volume (GMV) for brain structure, and resting-state functional connectivity (rsFC) and amplitude of low-frequency fluctuation (ALFF) for brain function. Additionally, event-related potential (ERP) P300 latency, a neuroelectrophysiological measure of cognitive processing speed obtained via electroencephalography (EEG), was assessed. All outcome measurements were conducted immediately following the intervention period. (5) Study design: We included only randomized controlled trials (RCTs) published in peer-reviewed journals.

### 2.3 Bias risk assessment

The risk of bias was evaluated by two researchers using the Cochrane bias risk Assessment tool (Cumpston et al., 2019). The assessment tool comprised six evaluation criteria encompassing selection bias (random sequence generation, assignment concealment), implementation bias, measurement bias, follow-up bias, reporting bias, and other bias. Each included literature was categorized as high risk, low risk, or unclear based on the degree of bias. In instances where researchers held conflicting views, resolution was achieved through discussion.

### 2.4 Data extraction

Data was extracted by two researchers according to study characteristics, such as publication year, sample size, age, gender, intervention duration, intervention frequency, mind–body exercise type of experimental group, control group, outcome indicators (MoCA, GMV, rsFC, ALFF, ERP P300 latency).

### 2.5 Statistical analysis

Data analysis was performed using Review Manager (RevMan) version 5.4. For continuous outcome measures, mean difference (MD) with 95% confidence interval was calculated when studies utilized identical assessment instruments. When different instruments were employed to evaluate the same construct, standardized mean difference (SMD) with 95% confidence interval was applied. A random-effects model was implemented for all analyses to account for potential methodological variations among included studies. Statistical heterogeneity was assessed using the Higgins I^2^ statistic, with values ranging from 75 to 100% indicating substantial heterogeneity (Higgins et al., 2008). When substantial heterogeneity was detected (I^2^ > 75%), subgroup analyses were conducted to explore potential influencing factors.

For neuroimaging outcomes, this study employed a descriptive summary and systematic mapping approach. Due to variations in neuroimaging acquisition parameters across included studies and the limited number of studies meeting the requirements for coordinate-based meta-analysis, formal meta-analytic methods such as activation likelihood estimation (ALE) were not employed. Specifically, we extracted three-dimensional coordinates of brain regions demonstrating significant between-group differences from the included studies. These coordinates were subsequently visualized on a standard brain template using BrainNet Viewer software, which displayed coordinate points integrating all significant clusters reported across the included studies, with the objective of providing a systematic visual synthesis of existing findings.

## 3 Results

### 3.1 Literature selection

**Figure 1:**
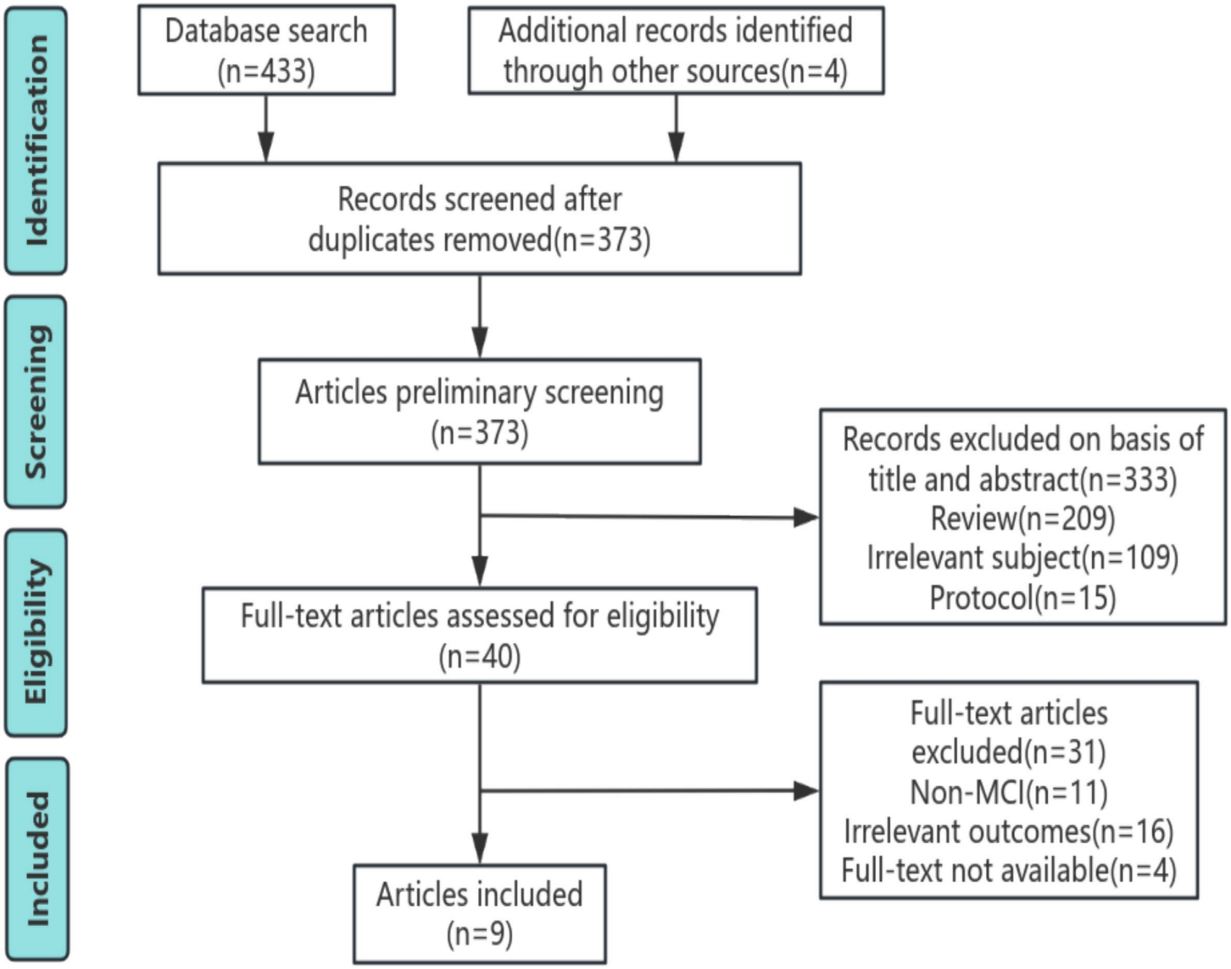
PRISMA diagram.

A preliminary database search yielded 433 studies and four articles were added from other sources. Following the removal of duplicate articles, 373 remained for further evaluation. Through title and abstract review, 334 articles were excluded, encompassing 209 reviews, 15 protocols, and 109 articles irrelevant to the subject. Full-text analysis was conducted on the remaining 39 articles, leading to the exclusion of 31 (11 lacked subjects with MCI, 16 did not report on brain function or structure, and 4 lacked usable data). Ultimately, 9 articles met the inclusion criteria. The detailed literature screening process is illustrated in Figure 1.

### 3.2 Research characteristics and results

In the 9 studies included (Eyre et al., 2016; Yang et al., 2016; Zhu et al., 2018, 2022; Qi et al., 2019; Tao et al., 2019; Xia et al., 2019; Liu et al., 2021; Zheng et al., 2021), all participants met the diagnostic criteria for MCI, totaling 462 individuals. The impact of mind–body exercise on the brain was primarily assessed through various methods as follows: (1) Cognitive performance assessment: Cognitive function was evaluated using neuropsychological tests, while global cognitive function was measured using MoCA in four studies listing post-treatment the scores (Zhu et al., 2018; Qi et al., 2019; Tao et al., 2019; Zheng et al., 2021). (2) Brain structure assessment: Five studies (Yang et al., 2016; Tao et al., 2019; Liu et al., 2021; Zheng et al., 2021; Zhu et al., 2022) reported changes in gray matter volume in different brain regions post-intervention. (3) Brain function assessment: Five studies (Eyre et al., 2016; Qi et al., 2019; Tao et al., 2019; Xia et al., 2019; Liu et al., 2021) assessed changes in resting-state functional connectivity using fMRI, while two studies (Qi et al., 2019; Liu et al., 2021) used ALFF to evaluate spontaneous brain activity. One study (Zhu et al., 2018) assessed neural activity in the brain through the latency of ERP P300. Another study (Yang et al., 2016) evaluated brain neurochemical plasticity through changes in brain metabolites. For more details, refer to Table 1.

**Table 1:** Characteristics of the included studies.

Study	Sample size	Intervention/control total (male: females)	Mean ± SD age, years	Intervention frequency	Assess time	Outcome measures	Main outcomes	
							Brain structure	Brain function
Zhu et al. (2018)	60	Dance:29(14:15) Usual care:31(10:21)	70.3 ± 6.7 69.0 ± 7.3	35 min 3times/week 12 weeks	Pre-intervention 3 months 6 months	P300 latency and amplitude	—	EEG: dance group showed a greater improvement in memory and processing speed (P300 latency changes) compared to control
Qi et al. (2019)	32	Dance:16(5:11) Usual care:16(4:12)	70.6 ± 6.2 69.1 ± 8.1	35 min 3times/week 12 weeks	Pre-intervention 3 months	ALFF	—	ALFF: dance increased ALFF values in the bilateral frontotemporal lobes, the insular cortex, the anterior cingulate gyrus, and the parahippocampal gyrus.
Yang et al. (2016)	25	Kundalini Yoga:14(8:6) Memory training:11(5:6)	67.1 ± 9.5 67.8 ± 9.7	60 min 1times/week 12 week	Pre-intervention 3 months	GMV, BMA	GMV: (1) Kundalini Yoga group: no changes in dACC or hippocampal gray matter volume were noted. (2) Memory training group: increased gray matter volume in dACC.	BMA: (1) Kundalini Yoga group: no change in the concentration of brain metabolites (2) Memory training group: decreased levels of choline in the bilateral hippocampus
Eyre et al. (2016)	25	Kundalini Yoga:14(8:6) Memory training:11(5:6)	67.1 ± 9.5 67.8 ± 9.7	60 min 1times/week 12 week	Pre-intervention 3 months	rsFC	—	rsFC: (1) In Kundalini Yoga group, improved verbal memory performance was observed to be correlated with increased connectivity between DMN and the frontal medial cortex, pregenual anterior cingulate cortex, right middle frontal cortex, posterior cingulate cortex, and left lateral occipital cortex, as well as correlated with increased connectivity between the language processing network and the left inferior frontal gyrus. (2) Improved visuospatial memory performance was inversely associated with connectivity between the superior parietal network and the medial parietal cortex in Kundalini Yoga group.
Tao et al. (2019)	57	Baduanjin:20(5:15) Brisk walking:17(7:10) Health education:20(6:14)	66.17 ± 4.17 64.32 ± 2.60 65.97 ± 5.66	60 min 3times/week 24 weeks	Pre-intervention 6 months	GMV, rsFC ALFF	GMV:(1) Baduanjin increased gray matter volume in the right hippocampus compared to brisk walking (2) Baduanjin increased gray matter volume in bilateral ACC compared to the health education group:	ALFF: a decrease in ALFF was observed in the right hippocampus and an increase in ALFF in the ACC and mPFC on both sides in the Baduanjin group. rsFC: an increase in rsFC was observed between the right hippocampus and the right angular gyrus in the Baduanjin group.
Liu et al. (2021)	57	Baduanjin:20(5:15) Brisk walking:17(7:10) Health education:20(6:14)	66.17 ± 4.17 64.32 ± 2.60 65.97 ± 5.66	60 min 3times /week 24 weeks	Pre-intervention 6 months	GMV, rsFC	GMV: Baduanjin increased gray matter volume in the right ACC compared to the other groups	rsFC: Baduanjin group showed the rsFC between LC-TPJ and VTA-TPJ increased
Xia et al. (2019)	69	Baduanjin:23(6:17) Brisk walking:23(11:12) Health education:23(6:17)	65.79 ± 4.35 64.88 ± 3.30 65.86 ± 5.28	60 min 3times/week 24 weeks	Pre-intervention 6 months	rsFC	**—**	rsFC: (1) DAN of Baduanjin exercise group exhibited functional connectivity decreased in right rolandic operculum, right middle temporal gyrus, right supramarginal inferior parietal, angular gyri, right precuneus, and right fusiform gyrus regions compared with the other two groups (2) Walking group: functional connectivity of the parietal inferior angular gyrus increased markedly, and the right middle temporal gyrus descended
Zheng et al. (2021)	69	Baduanjin:23(6:17) Brisk walking:23(11:12) Health education:23(6:17)	65.79 ± 4.35 64.88 ± 3.30 65.86 ± 5.28	60 min 3times/week 24 weeks	Pre-intervention 6 months	GMV	GMV: (1) Baduanjin increased gray matter volumes in the temporal lobe, frontal lobe, parietal lobe, medial occipital lobe, cingulate gyrus and angular gyrus compared to brisk walking. (2) Baduanjin increased gray matter volumes in the right frontal lobe, central anterior lobe, and occipital lobe compared to the health education	**—**
Zhu et al. (2022)	68	Dance:35(18:17) Control:33(23:10)	71.51 ± 6.62 69.82 ± 7.74	35 min 3times /week 12 weeks	Pre-intervention 3 months	GMV	GMV: Dance increase the right and total hippocampal volumes	**—**

rsFC, resting-state functional connectivity; ALFF, amplitude of low-frequency fluctuation; dACC, dorsal anterior cingulate gyrus; DMN, default mode network; mPFC, medial prefrontal cortex; LC, locus coeruleus; VTA, ventral tegmental area; TPJ, bilateral temporoparietal junction; DAN, dorsal attentional network; BMA, Brain Metabolite Analysis.

The clinical characteristics, training protocols, and outcomes of the nine randomized controlled trials (RCTs) analyzed are detailed in Table 1. In the experimental group interventions, three studies (Zhu et al., 2018, 2022; Qi et al., 2019) implemented specifically designed moderate-intensity aerobic dance programs, consisting of preparatory, aerobic dance, and finishing stages; two studies focused on yoga meditation training (Eyre et al., 2016; Yang et al., 2016); and four studies utilized Baduanjin, a traditional Chinese exercise (Tao et al., 2019; Xia et al., 2019; Liu et al., 2021). These various interventions constituted multi-component exercise training for the experimental group. Meanwhile, the control group received diverse interventions such as health education, daily care, memory training, and brisk walking. The intervention durations ranged from 3 to 6 months, with frequencies varying from 1 to 3 times per week, and session lengths lasting between 35 to 60 min. All studies conducted pre-and post-intervention assessments, with significant results observed in seven of the included studies.

**Figure 2:**
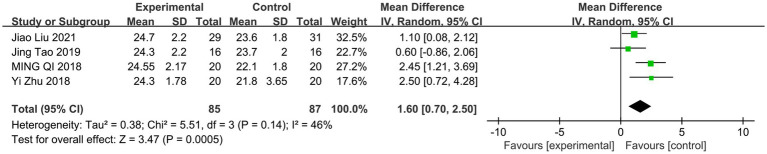
Forest plots of meta-analysis of primary outcomes.

This study conducted a meta-analysis of the effects of interventions for global cognitive function. As shown in Figure 2, a meta-analysis of 4 studies using the MoCA scale demonstrated a significant effect of aerobic exercise intervention on global cognitive function improvement (MD = 1.6; 95% CI: 0.70 to 2.50; *p* = 0.0005). Heterogeneity tests indicated moderate heterogeneity among the studies (I^2^ = 46%, τ^2^ = 0.38), which directly impacts the reliability of the pooled results. Given the limited number of included studies, it is difficult to trace the specific sources of heterogeneity, and thus subgroup analysis or other methods cannot be applied to identify its origins. Consequently, although the pooled effect size is statistically significant, the moderate heterogeneity suggests that the findings of this study should be interpreted as providing preliminary support for the benefits of mind–body exercises rather than conclusive evidence. These findings serve as an important summary of the current evidence and lay the groundwork for more in-depth future research (Zhu et al., 2018; Qi et al., 2019; Tao et al., 2019; Liu et al., 2021).

**Figure 3:**
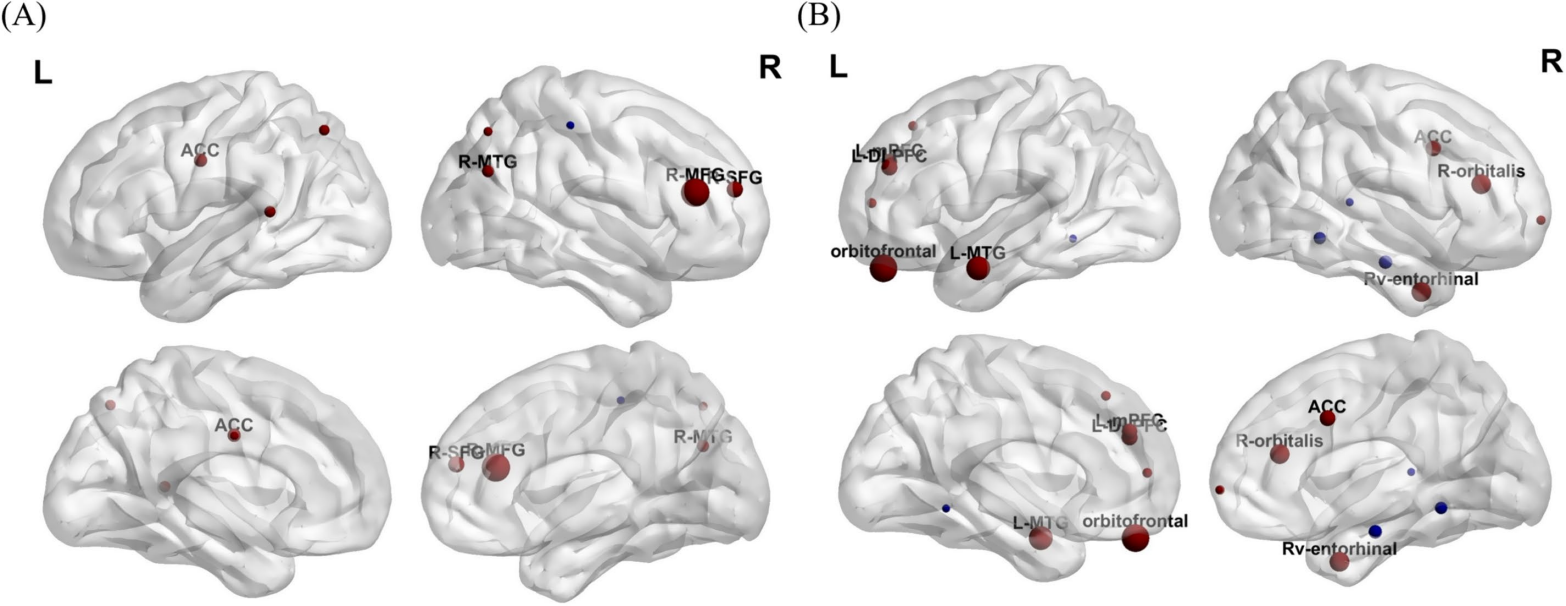
**(A)** Changes in gray matter volume in different brain regions after intervention. **(B)** Changes of ALFF in different brain regions after intervention. The size of the ball represents the size of the Cluster, which indicates the magnitude of significance (Red: increased ALFF, Blue: decreased ALFF). MFG, middle frontal gyrus; SFG, superior frontal gyrus; ACC, anterior cingulate cortex; MTG, middle temporal gyrus; DLPFC, dorsolateral prefrontal cortex; mPFC, medial prefrontal cortex; Rv-entorhinal, right ventral entorhinal cortex.

**Figure 4:**
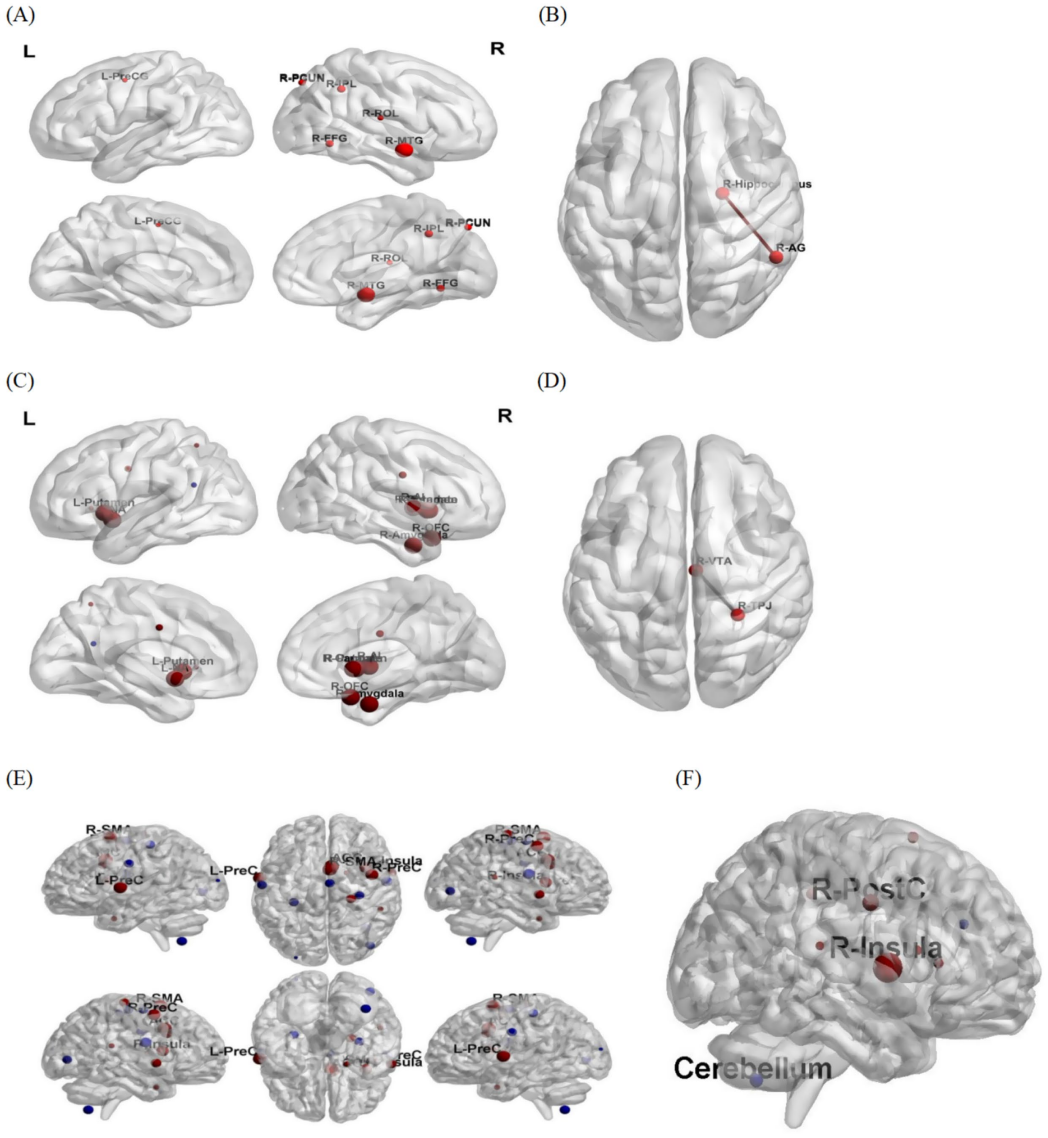
Brain regions exhibiting significant functional connectivity. **(A)** DAN as the seed point. **(B)** hippocampus as seed point. **(C)** The left VTA as seed point. **(D)** The right VTA as seed point. **(E)** The right locus coeruleus as the seed point. **(F)** The left locus coeruleus as the seed spot. The size of the ball represents the size of the Cluster, which indicates the magnitude of significance (Red: Enhanced functional connectivity, Blue: Weakened functional connectivity). PCUN, precuneus; IPL, inferior parietal lobule; ROL, Roland-Dick island gim; FFG, fusiform gyrus; Pre-CG, precentral gyrus; MFG, middle frontal gyrus; AG, angular gyrus; OFC, orbitofrontal gyrus amygdala; NA, nucleus accumbens; AI, anterior insula; TPJ, temporoparietal symphysi; SMA, Supplementary Motor Area; insula, insular cortex; PreC, Precentral gyrus; ACC, Anterior cingulate cortex; PostC, Postcentral gyrus cerebellum.

In the included studies, three-dimensional coordinates from imaging were extracted and plotted in the map. Figures 3, 4 illustrate the alterations in brain structure and function following interventions. Most studies have shown positive outcomes after interventions. Figure 4 displays brain regions with a significant increase in gray matter volume and alterations in ALFF post-intervention. Figure 4A shows the activation regions of functional connectivity with the dorsal attentional network (DAN) as a seed point, a circuit central to top-down attentional control and goal-directed behavior. Figure 4B exhibits enhanced functional connectivity between the right hippocampus and the right angular gyrus (R-AG) suggesting strengthened circuitry for episodic memory retrieval, a pathway critically involved in episodic memory retrieval and the integration of memory information. Figures 4C,D illustrate the functional connectivity based on bilateral ventral tegmental area (VTA) seed points. Figures 4E,F show the brain regions with changed functional connectivity seeded by the bilateral locus ceruleus, key neuromodulatory nuclei whose broad projections are fundamental to core cognitive processes such as attention, motivation, and cognitive control.

### 3.3 Bias risk assessment

**Figure 5:**
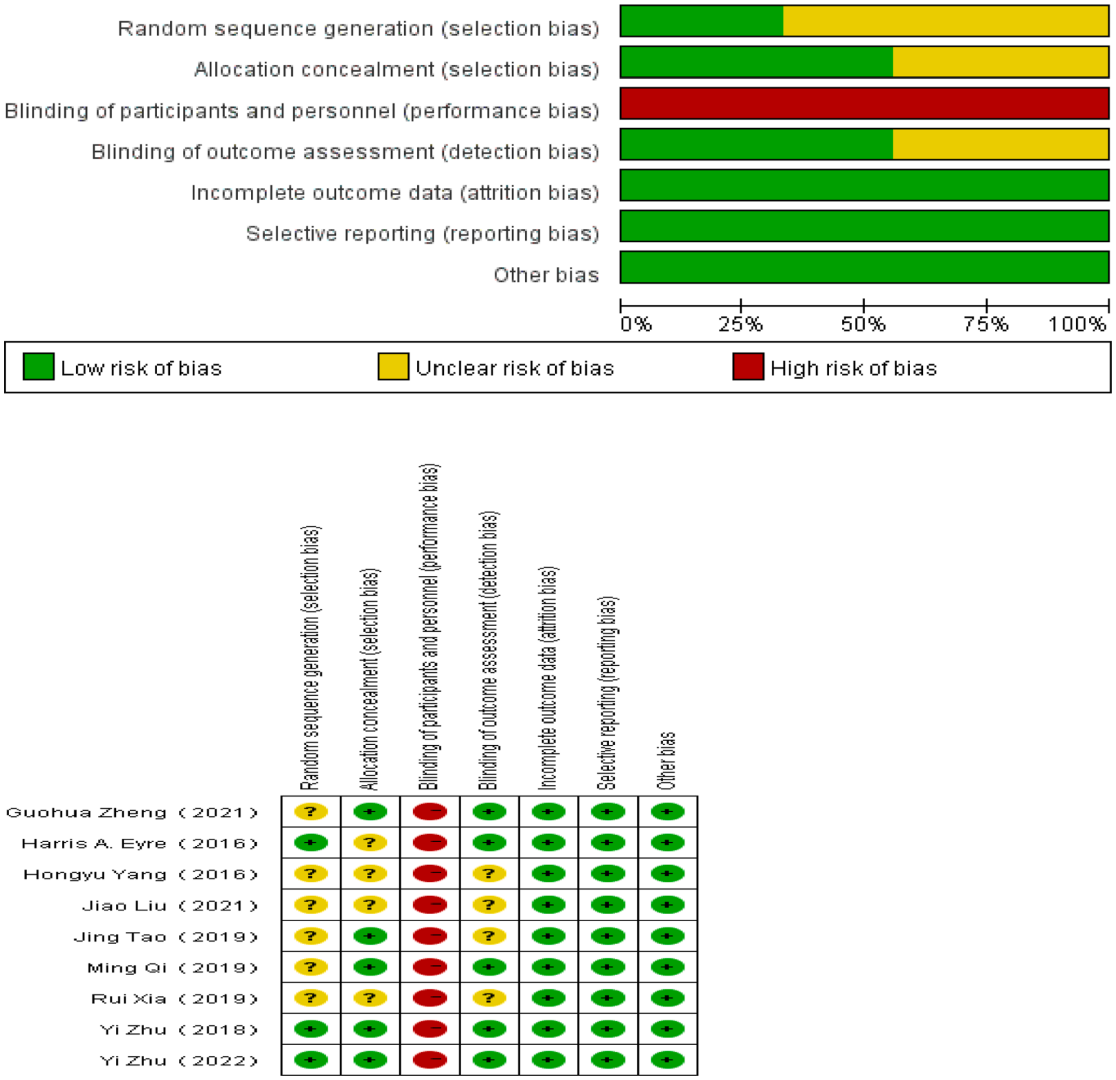
Risk of bias assessment.

The biases identified in the reviewed studies are illustrated in Figure 5. Several studies employed standard methods, such as computer-generated randomized sequences, for implementing allocation concealment strategies, resulting in the categorization of allocation concealment as a “low risk” level (Zhu et al., 2018, 2022; Qi et al., 2019; Tao et al., 2019; Zheng et al., 2021). However, other studies did not specify their allocation concealment methods, leading to an “unclear” status (Eyre et al., 2016; Yang et al., 2016; Xia et al., 2019; Liu et al., 2021). Due to the inherent characteristics of the intervention, the implementation of blinding, both for researchers and participants, was not feasible across all studies reviewed. In five studies, all result assessors were blinded (Eyre et al., 2016; Zhu et al., 2018, 2022; Qi et al., 2019; Zheng et al., 2021), whereas blinding status of assessors was unspecified in the remaining four studies (Yang et al., 2016; Tao et al., 2019; Xia et al., 2019; Liu et al., 2021). All studies were assessed as having a low risk of attrition bias and reporting bias.

## 4 Discussion

### 4.1 Summary of research results

This systematic review and meta-analysis, based on 9 randomized controlled trials, aimed to comprehensively evaluate the benefits of mind–body exercise for cognitive function and neuroplasticity in patients with mild cognitive impairment. A meta-analysis of the four included trials showed that combined physical and mental exercise improved global cognition in older adults with MCI (Zhu et al., 2018; Qi et al., 2019; Tao et al., 2019; Liu et al., 2021). Five studies reported significant gray matter volume increases in critical regions including the hippocampus, cingulate gyrus, frontal lobe, and temporal lobe (Yang et al., 2016; Tao et al., 2019; Liu et al., 2021; Zheng et al., 2021; Zhu et al., 2022). The six studies collectively documented functional alterations, as evidenced by changes in the connectivity of the default mode network (DMN), DAN and language networks, significant fluctuations in ALFF, and a marked decrease in ERP P300 latency (Eyre et al., 2016; Zhu et al., 2018; Qi et al., 2019; Tao et al., 2019; Xia et al., 2019; Liu et al., 2021). Conversely, yoga-focused interventions showed no significant structural or metabolic alterations (Yang et al., 2016).

### 4.2 The effects of mind–body exercise on cognitive function

The meta-analysis of four randomized controlled trials revealed that mind–body exercise was associated with a statistically significant improvement in MoCA scores (MD = 1.6; 95% CI: 0.70 to 2.50; *p* = 0.0005). This observed improvement suggests that some patients’ overall cognitive levels may shift toward the normal range, which holds positive implications for maintaining their independence in daily activities. The cognitive benefits were consistent with enhanced information processing speed, as reflected by shortened P300 latency following the intervention, further supporting its potential value in improving the efficiency of performing daily tasks. Notably, these improvements were partially sustained for up to 5 months after the intervention concluded, indicating that the positive effects of mind–body exercise are not only immediate but may also continuously slow the natural progression of cognitive decline, thereby supporting long-term functional independence and quality of life in patients.

While the meta-analysis suggests potential cognitive improvement, the results across individual studies were inconsistent, which appears to be related to intervention type and dosage - the primary source of heterogeneity among the included studies. In terms of intervention type, aerobic dance emphasizes cardiovascular exercise and rhythmic movement memorization, whereas Baduanjin integrates slow movements, breath control, and meditation. These fundamental differences in motor characteristics and cognitive engagement may engage partially distinct neural mechanisms, thereby constituting an important source of outcome variation (Voelcker-Rehage and Niemann, 2013). Regarding duration and intensity, two dance intervention studies (3 sessions/week, 35 min/session for 3 months) found no significant MoCA score improvements (Zhu et al., 2018; Qi et al., 2019). Conversely, two studies investigating the Baduanjin exercise regimen, conducted thrice weekly for 60 min over 6 months, demonstrated significant cognitive enhancements (Tao et al., 2019; Liu et al., 2021). This suggests that an adequate cumulative dosage may be a necessary condition for eliciting measurable cognitive benefits, a finding consistent with other exercise studies (Erickson et al., 2019; Huang et al., 2022). These differences in intervention parameters directly explain the inconsistencies in results across studies and further support the cautious stance we maintain in the meta-analysis.

Heterogeneity was observed across key intervention parameters, specifically in dosage (session duration, frequency, total length) and in the level of supervision (ranging from professional guidance to self-directed practice). These factors represent critical moderators of treatment effects. Evidence suggests that these variables, particularly supervision quality, significantly shape treatment efficacy by influencing participant adherence and movement precision (Ning et al., 2024; Li et al., 2025). Due to the limited reporting details and number of original studies, we could not perform subgroup analyses; consequently, this variability limits the generalizability of any conclusions regarding an optimal intervention protocol. The pooled results should therefore be interpreted as an overall effect estimate of mind–body exercises across varying implementation conditions. Future research should focus on standardizing intervention protocols and systematically assessing implementation factors such as supervision.

### 4.3 The effects of mind–body exercise on brain structure

Our results align with ten Brinke et al. (2015) and Ribeiro et al. (2025) confirming that exercise effectively increases hippocampal volume - a crucial memory-related region in MCI patients. Notably, In this review, the research on Baduanjin not only replicated the hippocampal effect but also consistently showed a significant increase in the volume of ACC (Tao et al., 2019; Liu et al., 2021; Zheng et al., 2021). These findings suggest that mind–body exercises like Baduanjin may uniquely target the ACC, a region governing executive function and emotion regulation.

This systematic review identifies a pattern where Baduanjin is linked to simultaneous volume increases in the hippocampus, ACC, and distributed cortical gray matter (Tao et al., 2019; Zheng et al., 2021; Zhu et al., 2022), exhibiting a more extensive neuroanatomical impact than the hippocampal-frontal pattern characteristic of traditional aerobic exercise (Erickson et al., 2019; Frost et al., 2022). These comprehensive neuroplastic changes likely originate from Baduanjin’s integrative approach combining aerobic activity, motor learning, mindfulness practice, and respiratory regulation, which synergistically generate enhanced multisensory integration and cognitive engagement.

Notably, the included studies revealed no significant structural brain changes from yoga interventions in MCI patients (Yang et al., 2016), contrasting with reported neuroanatomical effects in healthy older adults and chronic disease populations. This discrepancy may arise from three key factors which require further empirical testing: (1) unique neuropathological characteristics of MCI populations, (2) variability in intervention protocols (including dosage, intensity, and yoga style), and (3) yoga’s potentially stronger influence on functional connectivity than gross morphological changes (Johnen et al., 2015).

Our neuroimaging analysis revealed right-hemisphere predominant volumetric increases in the hippocampus (Zhu et al., 2022), ACC (Tao et al., 2019; Liu et al., 2021), and frontal lobe (Zheng et al., 2021). This lateralized pattern corresponds with Thompson et al.’s (Thompson et al., 2003) well-documented findings of right-sided vulnerability in early-stage AD pathology, particularly within hippocampal-temporal circuits. These observations suggest that mind–body exercise, particularly Baduanjin, may preferentially enhance neuroplastic adaptation or confer neuroprotection in these right-lateralized regions exhibiting early pathological susceptibility.

### 4.4 The effects of mind–body exercise on brain function

While the exact mechanisms remain to be fully elucidated, our findings, combined with existing literature, suggest several potential pathways through which mind–body exercise may influence brain function.

The aging brain maintains cognitive function through compensatory mechanisms that offset structural decline with functional network reorganization (Grady, 2012). Our meta-analysis uncovers disparate neurocognitive activation patterns across different interventions: (1) Dance predominantly involves frontal cortical regions linked to executive function, memory integration, and motor planning; (2) Baduanjin elicits a more targeted modulation of the DMN, DAN and neurotransmitter systems; (3) whereas yoga did not demonstrate significant metabolic changes in the present analysis.

The PCC/precuneus, serving as the central hub of the DMN, exhibits particular vulnerability to functional connectivity degradation during cognitive decline. Notably, a 12-week walking intervention enhanced PCC/precuneus-hippocampal connectivity in individuals with MCI (Buckner et al., 2008; Chirles et al., 2017). Consistent with these findings, yoga practice has been shown to strengthen DMN connectivity and enhance verbal memory performance, despite demonstrating no significant effects on cerebral metabolite levels.

The observed enhancement in functional connectivity between the locus coeruleus (LC) /VTA and bilateral temporoparietal junction (TPJ) as well as the insula following Baduanjin practice may reflect modulation of neurotransmitter systems involved in alertness, motivation, and stimulus-driven attention, potentially through noradrenergic/dopaminergic pathways (Liu et al., 2021). This neural mechanism aligns with behavioral evidence from Rui Xia et al. showing improved selective attention following Baduanjin practice (Xia et al., 2019). Animal studies further substantiate these effects, demonstrating that: (1) physical exercise enhances recognition memory via LC activation in rodent models (Sara, 2009), and (2) increases prefrontal dopamine and serotonin (5-HT) levels while restoring synaptic plasticity (Lima et al., 2023).

ALFF reflect both neural degeneration and compensatory neuroplasticity. In individuals with MCI, hippocampal hyperactivation may represent a compensatory response to structural atrophy, offering potential for early MCI detection (Xi et al., 2012; Wang et al., 2018; Zhang et al., 2024). Our systematic review found that Baduanjin improves cognitive function through a dual mechanism: increasing hippocampal volume and reducing ALFF abnormalities by correcting abnormal hippocampal activity (Tao et al., 2019). In contrast, aerobic dance is associated with more widespread ALFF increases across multiple regions, including bilateral frontotemporal cortices, entorhinal cortex, and anterior cingulate cortex - likely reflecting its greater cognitive-motor integration demands (Qi et al., 2019). Notably, dance interventions also shorten P300 latency and accelerate information processing speed in MCI patients, with these benefits persisting at 5-month follow-up (Zhu et al., 2018). These findings underscore the importance of sustained, moderate-intensity dance training for maintaining cognitive improvements.

### 4.5 Limitations and future directions

This review has several methodological limitations to consider. First, although we searched four major databases, our restriction to English publications may have introduced language bias. Second, our specific focus on neuroimaging outcomes in MCI populations following mind–body exercise substantially limited the number of eligible studies, consequently restricting the review’s scope and statistical power. Third, the observed sex distribution imbalances in some included studies may affect the generalizability of our findings. The absence of sex-stratified analyses in the original studies prevented assessment of potential sex-specific effects. In addition, population heterogeneity from varied MCI criteria and age range cautions against overgeneralization of the results. Finally, the available literature lacks integration with molecular-level data, which hampers a deeper understanding of the mechanisms underlying mind–body exercise induced neuroplastic changes.

Future research should prioritize large-scale, sex-matched randomized controlled trials that combine neuroimaging with relevant molecular biomarkers including BDNF, inflammatory cytokines, and Alzheimer-related proteins such as Aβ and tau. Such multimodal approaches will be essential to elucidate the underlying mechanisms and validate the effects of mind–body exercise across diverse demographic and cognitive populations.

## 5 Conclusion

This systematic review and meta-analysis demonstrates that mind–body exercise is associated with improvements in global cognitive function in older adults with MCI. The systematic mapping of neuroimaging evidence further indicates that structural and functional changes consistently involve specific brain regions, with the hippocampus and anterior cingulate cortex being the most frequently reported. These findings synthesize behavioral outcomes with neuroimaging data, providing converging evidence for the benefits of mind–body exercise in MCI.

## Data Availability

The data analyzed in this study is subject to the following licenses/restrictions: the data supporting this study’s findings are available from the corresponding author upon reasonable request. Requests to access these datasets should be directed to HT tianhuif1029@163.com.
