# Correction: The effect of mind–body exercise on cognitive function and neuroplasticity in elderly people with mild cognitive impairment: a systematic review and meta-analysis

**DOI:** 10.3389/fnagi.2026.1772273

**Published:** 2026-02-11

**Authors:** Huifang Tian, Xi Yang, Jiahuan Li, Yuqi Cheng, Shui Tian, Fanfan Meng, Qinqin Zhu, Ying Shen, Tong Wang, Chuan Guo, Yi Zhu

**Affiliations:** 1 Department of Rehabilitation Medicine, The First Affiliated Hospital of Nanjing Medical University, Nanjing, China; 2 The Fourth Affiliated Hospital of Soochow University (Suzhou Dushu Lake Hospital), Suzhou, China; 3 Department of Radiology, The First Affiliated Hospital of Nanjing Medical University, Nanjing, China

**Keywords:** mind–body exercise, mild cognitive impairment, gray matter volume, resting state magnetic resonance, event-related potential

In the published article, the figures were in the erroneously ordered and did not reflect the intended sequence. The images that should have appeared as Figure 1 and Figure 2 were displayed as Figure 3 and Figure 4, respectively (and vice versa). The caption texts are accurate, but due to this mislabeling, the order in which the figures appeared did not correspond to the sequence in which they were cited in the text. The order has now been corrected.

**Figure 1:**
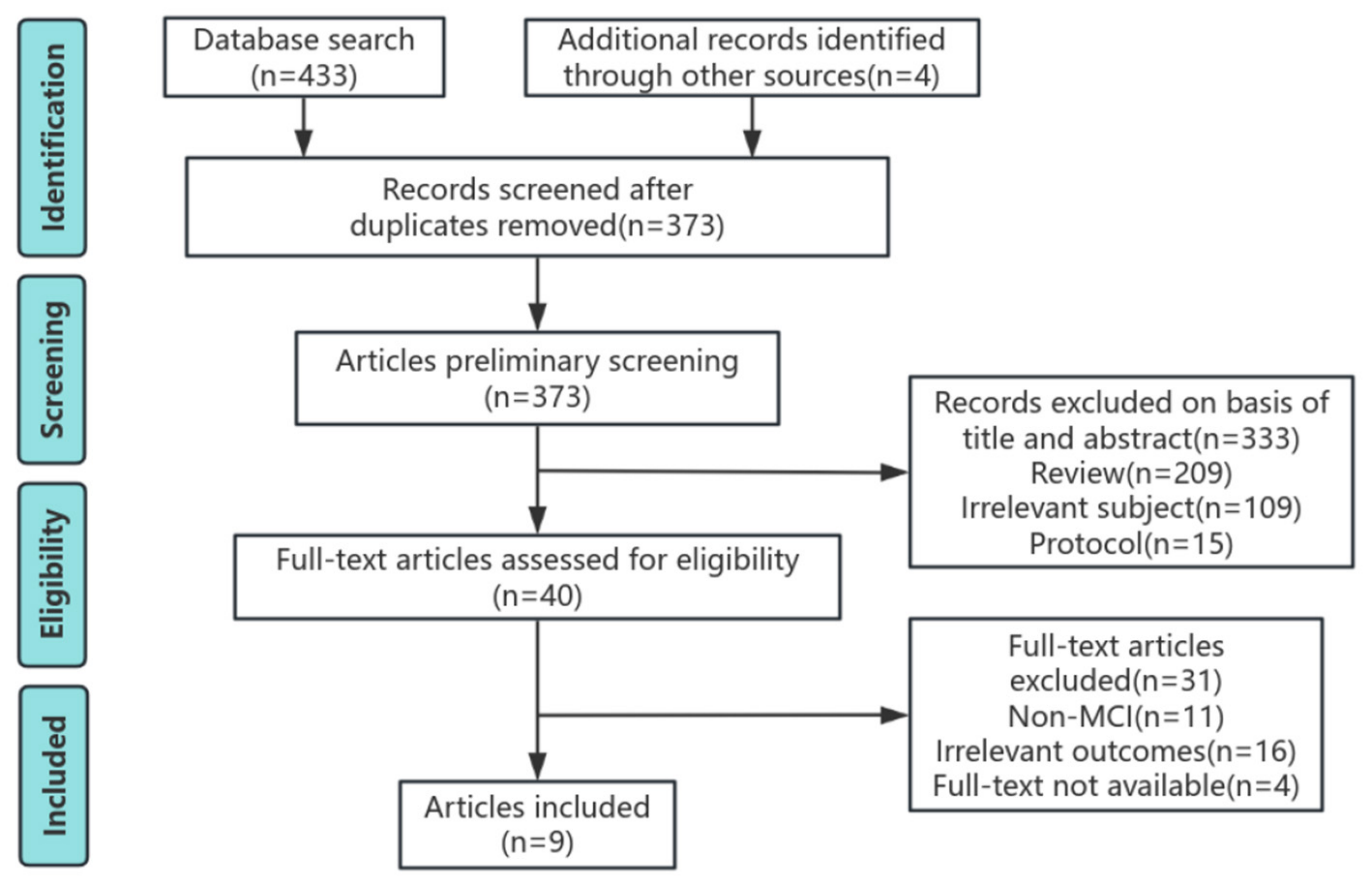
PRISMA diagram.

**Figure 2:**
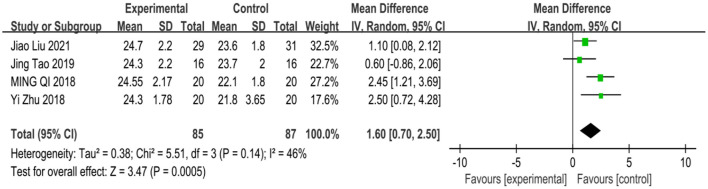
Forest plots of meta-analysis of primary outcomes.

**Figure 3:**
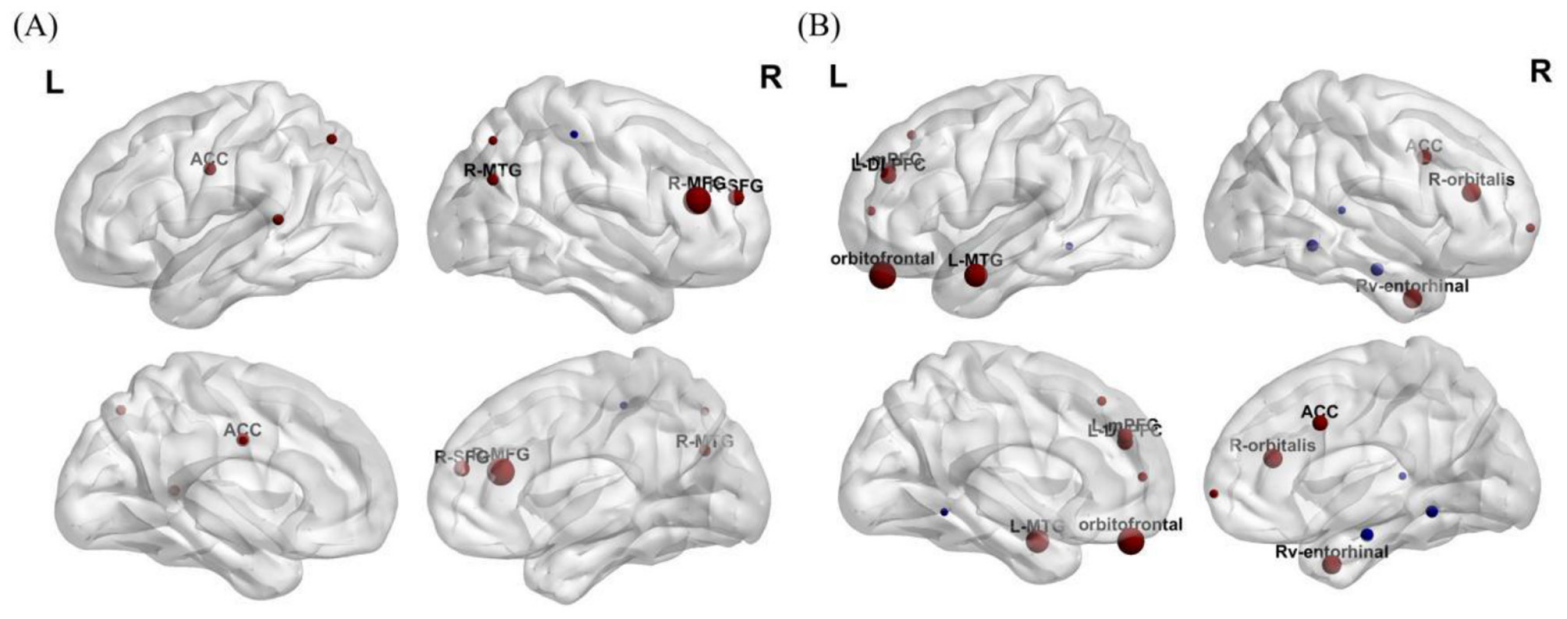
**(A)** Changes in gray matter volume in different brain regions after intervention. **(B)** Changes of ALFF in different brain regions after intervention. The size of the ball represents the size of the Cluster, which indicates the magnitude of significance (Red: increased ALFF, Blue: decreased ALFF). MFG, middle frontal gyrus; SFG, superior frontal gyrus; ACC, anterior cingulate cortex; MTG, middle temporal gyrus; DLPFC, dorsolateral prefrontal cortex; mPFC, medial prefrontal cortex; Rv-entorhinal, right ventral entorhinal cortex.

**Figure 4:**
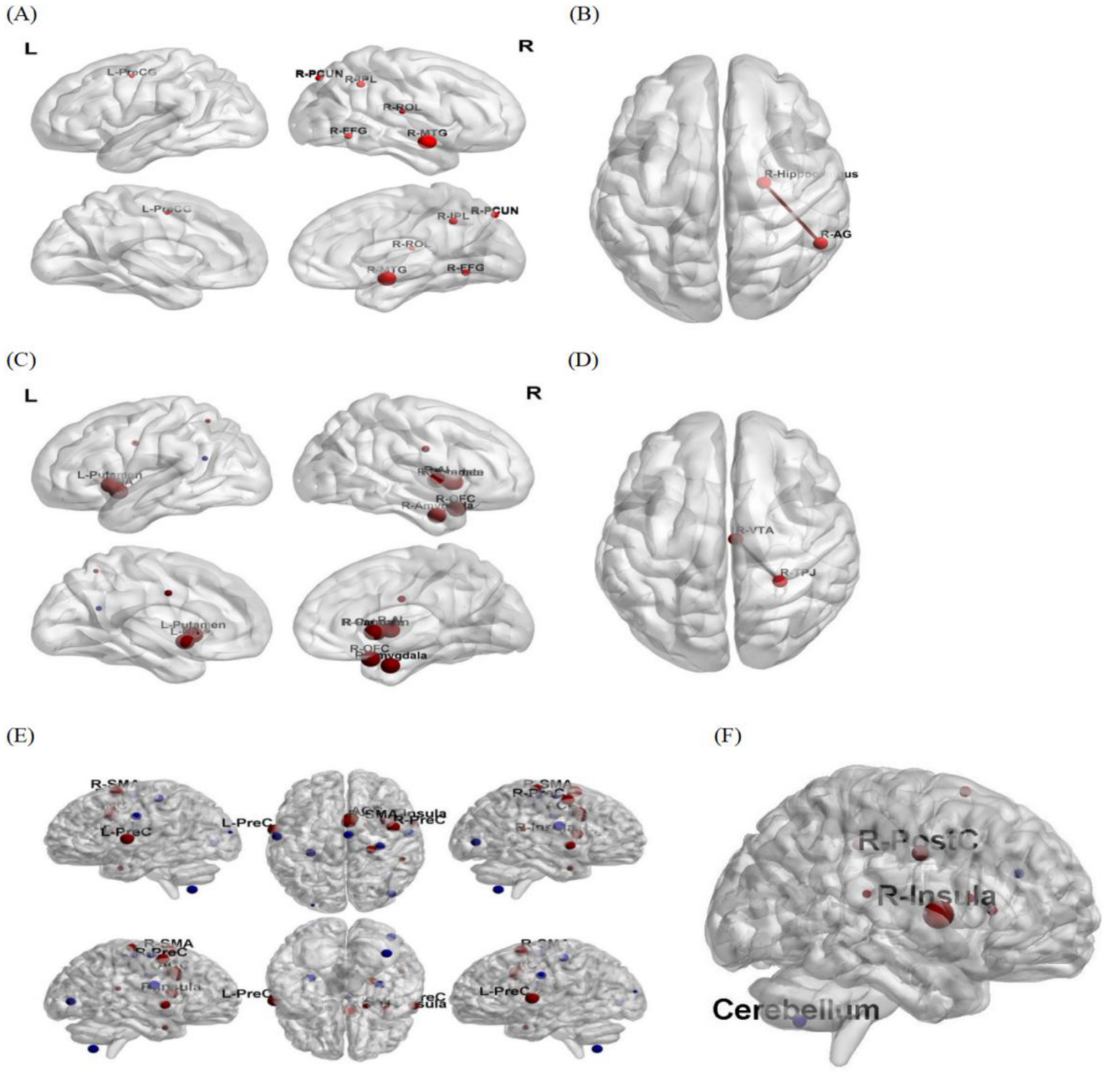
Brain regions exhibiting significant functional connectivity. **(A)** DAN as the seed point. **(B)** hippocampus as seed point. **(C)** The left VTA as seed point. **(D)** The right VTA as seed point. **(E)** The right locus coeruleus as the seed point. **(F)** The left locus coeruleus as the seed spot. The size of the ball represents the size of the Cluster, which indicates the magnitude of significance (Red: Enhanced functional connectivity, Blue: Weakened functional connectivity). PCUN, precuneus; IPL, inferior parietal lobule; ROL, Roland-Dick island gim; FFG, fusiform gyrus; Pre-CG, precentral gyrus; MFG, middle frontal gyrus; AG, angular gyrus; OFC, orbitofrontal gyrus amygdala; NA, nucleus accumbens; AI, anterior insula; TPJ, temporoparietal symphysi; SMA, Supplementary Motor Area; insula, insular cortex; PreC, Precentral gyrus; ACC, Anterior cingulate cortex; PostC, Postcentral gyrus cerebellum.

The original version of this article has been updated.

